# Modelling the widespread effects of TOC1 signalling on the plant circadian clock and its outputs

**DOI:** 10.1186/1752-0509-7-23

**Published:** 2013-03-19

**Authors:** Alexandra Pokhilko, Paloma Mas, Andrew J Millar

**Affiliations:** 1 School of Biological Sciences, University of Edinburgh, Mayfield Road, Edinburgh, EH9 3JH, UK; 2 Centre for Research in Agricultural Genomics (CRAG), Consortium CSIC-IRTA-UAB-UB, Parc de Recerca UAB, Bellaterra (Cerdanyola del Vallés), 08193, BarcelonaSpain; 3 SynthSys, University of Edinburgh, C.H. Waddington Building, Mayfield Road, Edinburgh, EH9 3JD, UK; 4 Institute for Complex Systems and Mathematical Biology, University of Aberdeen, Meston Building, Aberdeen, AB24 3UE, UK

**Keywords:** Circadian rhythms, Biological clocks, Gene regulatory networks, Mathematical model, Systems biology

## Abstract

**Background:**

24-hour biological clocks are intimately connected to the cellular signalling network, which complicates the analysis of clock mechanisms. The transcriptional regulator TOC1 (TIMING OF CAB EXPRESSION 1) is a founding component of the gene circuit in the plant circadian clock. Recent results show that TOC1 suppresses transcription of multiple target genes within the clock circuit, far beyond its previously-described regulation of the morning transcription factors LHY (LATE ELONGATED HYPOCOTYL) and CCA1 (CIRCADIAN CLOCK ASSOCIATED 1). It is unclear how this pervasive effect of TOC1 affects the dynamics of the clock and its outputs. TOC1 also appears to function in a nested feedback loop that includes signalling by the plant hormone Abscisic Acid (ABA), which is upregulated by abiotic stresses, such as drought. ABA treatments both alter TOC1 levels and affect the clock’s timing behaviour. Conversely, the clock rhythmically modulates physiological processes induced by ABA, such as the closing of stomata in the leaf epidermis. In order to understand the dynamics of the clock and its outputs under changing environmental conditions, the reciprocal interactions between the clock and other signalling pathways must be integrated.

**Results:**

We extended the mathematical model of the plant clock gene circuit by incorporating the repression of multiple clock genes by TOC1, observed experimentally. The revised model more accurately matches the data on the clock’s molecular profiles and timing behaviour, explaining the clock’s responses in *TOC1* over-expression and *toc1* mutant plants. A simplified representation of ABA signalling allowed us to investigate the interactions of ABA and circadian pathways. Increased ABA levels lengthen the free-running period of the clock, consistent with the experimental data. Adding stomatal closure to the model, as a key ABA- and clock-regulated downstream process allowed to describe TOC1 effects on the rhythmic gating of stomatal closure.

**Conclusions:**

The integrated model of the circadian clock circuit and ABA-regulated environmental sensing allowed us to explain multiple experimental observations on the timing and stomatal responses to genetic and environmental perturbations. These results crystallise a new role of TOC1 as an environmental sensor, which both affects the pace of the central oscillator and modulates the kinetics of downstream processes.

## Background

Circadian clocks allow most eukaryotes and some prokaryotes to anticipate the environmental day/night cycle, through rhythmic modulation of multiple physiological processes [1]. Circadian clocks are characterised by free running rhythms with a period of ~24 h even in the absence of any environmental cues, such as in constant light conditions. Their timing is synchronised with the environmental day-night cycle principally by responses to light and temperature, but clock circuits also respond to many additional stimuli. The signalling pathways from such stimuli are often found to be rhythmically controlled by the clock, forming nested feedback loops that modify the circadian oscillator. The gene circuit of the higher plant clock consists of interlocked morning and evening loops operating through negative feedback mechanisms [2]. TOC1 is an important component of the plant clock since its mutation or overexpression dramatically change the properties of the central oscillator [3].

Although the importance of TOC1 in the plant circadian clock has long being recognized, the precise role of TOC1 in the clock has been a matter of debate [2-7]. Based on indirect genetic evidence it was first proposed that TOC1 activated the expression of the key transcription factors *LHY* and *CCA1*[4], although several observations on clock mutants remained paradoxical. Recent experimental and computational data [2,3,7] have demonstrated that TOC1 functions as a repressor rather than an activator. The studies also show that TOC1 repression is not limited to CCA1 and LHY but to nearly all of the genes at the core of the oscillator [3]. The observed reduction in the expression of multiple clock genes by up-regulation of TOC1 could not be described by our previous model, where only *LHY* and *CCA1* were affected by TOC1 [2,3]. Here, we extend our previous model by including the repression of multiple clock genes by TOC1 and explore TOC1 effect on the clock. In particular, we analysed clock dynamics in various TOC1-misexpressed lines and addressed the long-standing paradox of simultaneous reduction of *LHY* and *CCA1* amplitudes in both *toc1* mutant and TOC1-overexpressed (*TOC1-ox*) plants [7,8].

The recently discovered pervasive negative effect of TOC1 on gene expression [3,7], the overlap between microarrays of the TOC1- and ABA-regulated genes and the gated induction of TOC1 by the stress hormone abscisic acid ABA [9] suggests that TOC1 might integrate environmental and circadian information to regulate downstream physiology at specific time of the day [9]. Here we combined the clock model with key processes regulated by ABA and explored mutual interactions between clock and ABA pathways. The model describes the regulation of stomatal aperture, one of the most important outputs of ABA signalling, which is gated by the clock [9]. Being a sensor of water availability in plants, ABA level increases under dry conditions, which accelerates closing of stomata in the afternoon [9-11]. In agreement with the microarray studies, TOC1 mis-expressing plants were shown to have altered ABA-dependent stomata closure and affected responses to drought conditions [9]. To model the observed gating of stomatal opening by the clock, we included the interaction of ABA with the ABAR protein (also known as the H subunit of Mg-chelatase, CHLH or GUN5) – a key component of the ABA signalling pathway, arguably postulated as ABA receptor [12]. Besides being one of the downstream targets of TOC1, ABAR was shown to be a key component in the gating of ABA signalling by the clock [9,13]. We used the model to explore the dynamics of the opening of stomatal pore under various genetic and environmental perturbations, such as mutations of clock genes and by changing the light and water conditions, and showed that the model provides good match to existing experimental data. Our results show how the dual roles of TOC1 are integrated, combining timing and environmental information to modulate downstream physiology.

## Results and discussion

### Model description

**Figure 1:**
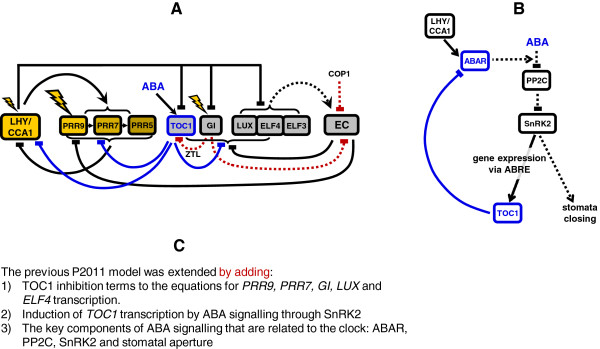
**Extended model of the plant circadian clock, including the multiple targets of TOC1 and the interactions with the ABA signalling pathway. A**: The clock model was extended from the previous one (P2011) by including the negative transcriptional regulations of the core clock genes by TOC1 (blue lines) and the up-regulation of *TOC1* expression by ABA signalling, as shown in panel B. Elements of the morning and evening loops are shown in yellow and grey, respectively. Proteins are shown only for EC, ZTL and COP1 for simplicity. Post-translational regulation of TOC1 and the EC by GI, ZTL and COP1 is indicated by the dotted red lines. **B**: Simplified model of ABA signalling, gated by the clock through regulation of *ABAR* expression. See section “Model description” for more details. Transcriptional and protein regulation are shown by solid and dotted lines, respectively. **C**: Short description of the newly-introduced connections and elements of the model compared to P2011.

The latest plant clock model [2], herein called P2011, was revised by adding the recently discovered negative regulation of the oscillator genes by TOC1 (blue lines on Figure 1A) as described in the section 1 below. Additionally, we have included a simplified version of the main steps leading to the induction of *TOC1* by ABA and the regulation of stomata aperture (Figure 1B), described in the section 2 below.

1. Extending the clock model by inhibition of gene expression by TOC1

**Figure 2:**
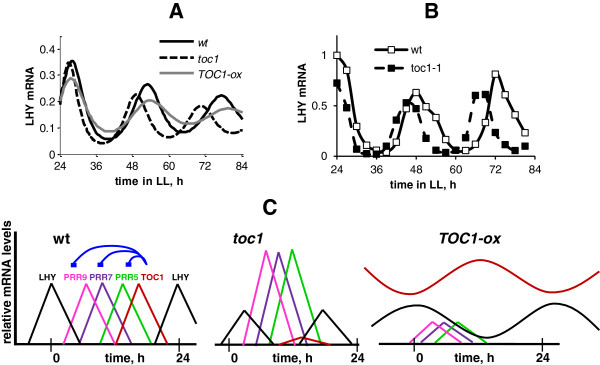
**Clock gene expression profiles in *TOC1 *mis-expressed lines. A**: The simulated profiles of *LHY/CCA1* mRNA under constant light conditions are shown for wild type (wt), *toc1* and TOC1-ox lines by black solid, dashed and grey lines, respectively. *TOC1-ox* was simulated with n16 = 0.1. Simulations were run after entrainment of the clock to 12 L:12D conditions to match the data. **B**: Experimental data for *LHY* mRNA in wild type and *toc1* plants under constant light conditions are redrawn from [4]; **C**: Cartoon illustrating the clock gene profiles in *TOC1* mis-expressed lines relative to the wild type. Coloured lines represent the expression of *LHY* and its inhibitors *PRR9, PRR7, PRR5, TOC1,* as shown in the legend for the wild type. Blue connections on the wild-type panel (left) show the inhibition of *PRR9, PRR7, PRR5* by TOC1. The release of this inhibition in the *toc1* mutant (centre) results in the increase in *PRR* expression, which causes the reduction of the LHY amplitude and, together with the absence of TOC1, shortens the clock period in the *toc1* mutant. On the contrary, overexpression of *TOC1* (right) reduces *PRR* expression and allows *LHY* levels to rise in the morning, while higher TOC1 delays *LHY* at the end of the night, lengthening the period in *TOC1-ox*.

The latest model of the Arabidopsis oscillator [2] was extended based on our recent data on the inhibition of expression of multiple clock genes by TOC1 and induction of *TOC1* through ABA signalling. The model is described by a system of 32 ODEs. The basic structure of the plant clock was kept from our previous P2011 model [2]. Briefly, the key components of the clock are the *LHY* and *CCA1* morning genes and the evening complex (EC) genes. *LHY* and *CCA1* are similarly expressed around dawn and act together to regulate multiple targets. They are described by single variable *LHY/CCA1* as before. EC (EVENING COMPLEX) genes *LUX*, *ELF3 (EARLY FLOWERING 3)* and *ELF4* are expressed around dusk and form the EC protein complex, which suppresses expression of multiple genes at night (Figure 1; [2]). *LHY/CCA1* expression is suppressed by the PRR proteins in a morning loop, while the EC is negatively regulated by the ubiquitin E3 ligase COP1 (CONSTITUTIVE PHOTOMORPHOGENIC 1), which targets ELF3 protein to degradation by proteasome and by GI protein, in the evening loop. The morning and evening loops are further interlocked through the suppression of evening gene expression by *LHY/CCA1* and suppression of *PRR9* and *TOC1 (PRR1)* expression by the EC. The model also includes the F box protein ZTL (ZEITLUPE), which negatively regulates the level of TOC1 protein. Light entrains the clock similarly to the P2011 model through several mechanisms, which are supported by experimental data [2]. The main mechanisms include acute activation of *LHY/CCA1*, *PRR9* and *GI* transcription (eqs. 1, 5, 27) immediately after dawn; stabilization of PRR proteins in presence of light (eqs. 6, 8, 10) and light-dependent regulation of the EC by COP1 and GI proteins (eqs. 24, 21, 22).

The TOC1-related reactions were substantially extended. Firstly, we added multiple reactions of inhibition of clock gene expression by TOC1. This includes direct inhibition of expression of *LHY/CCA1* as in the P2011 model (eq. 1)*,* and also *PRR9, PRR7, PRR5, LUX, ELF4* and *GI* (eqs. 5, 7, 9, 18, 14, 27), which is based on our data [3] (Figure 1A). The details of TOC1 interactions with other regulators (e.g. LHY/CCA1, EC, protein P) to modulate gene expression are largely unknown, so we assumed that TOC1 acts as a non-competitive inhibitor of gene expression Secondly, we added physiologically relevant activation of *TOC1* expression by the ABA signalling pathway (Figure 1B, [9]), described in section 2 below. This provided an additional level of input to the clock through ABA, which is induced by stress. Thirdly, we added regulation of stomata aperture as a clock output, which is directly related to both ABA and TOC1 signalling (Figure 1B), as presented in section 2 below.

The model is described by the following system of ODEs:

(1) $$\begin{array}{ll} \frac{dc_{L}^{m}}{\mathrm{dt}} & q_{1}Lc_{P}\\ & +n_{1}\frac{g_{1}{}^{a}}{g_{1}{}^{a}+{\left(c_{P9}+c_{P7}+c_{P5}+c_{T}\right)}^{a}}\\ & -\left(m_{1}L+m_{2}D\right)\cdot c_{L}^{m} \end{array}$$

(2) $$\frac{dc_{L}}{\mathrm{dt}}=\left(p_{2}+p_{1}L\right)\cdot c_{L}^{m}-m_{3}c_{L}-p_{3}\frac{c_{L}{}^{c}}{c_{L}{}^{c}+g_{3}{}^{c}}$$

(3) $$\frac{dc_{L\mathrm{mod}}}{\mathrm{dt}}=p_{3}\frac{c_{L}{}^{c}}{c_{L}{}^{c}+g_{3}{}^{c}}-m_{4}c_{L\mathrm{mod}}$$

(4) $$\frac{dc_{P}}{\mathrm{dt}}=p_{7}D\cdot \left(1-c_{P}\right)-m_{11}c_{P}L$$

(5) $$\begin{array}{ll} \frac{dc_{P9}^{m}}{\mathrm{dt}}= & \frac{g_{18}{}^{g}}{g_{18}{}^{g}+c_{T}{}^{g}}\\ & \times \left(L\cdot q_{3}\cdot c_{P}+\frac{g_{8}}{g_{8}+c_{\mathrm{EC}}}\left(n_{4}+n_{7}\cdot \frac{c_{L}{}^{e}}{g_{9}{}^{e}+c_{L}{}^{e}}\right)\right)\\ & -m_{12}c_{P9}^{m} \end{array}$$

(6) $$\frac{dc_{P9}}{\mathrm{dt}}=p_{8}c_{P9}^{m}-\left(m_{13}+m_{22}D\right)\cdot c_{P9}$$

(7) $$\begin{array}{ll} \frac{dc_{P7}^{m}}{\mathrm{dt}}= & \frac{g_{22}{}^{g}}{g_{22}{}^{g}+c_{T}{}^{g}}\\ & \times \left(n_{8}\frac{c_{\mathrm{Ltot}}{}^{e}}{g_{10}{}^{e}+c_{\mathrm{Ltot}}{}^{e}}+n_{9}\frac{c_{P9}{}^{f}}{g_{11}{}^{f}+c_{P9}{}^{f}}\right)\\ & -m_{14}c_{P7}^{m} \end{array}$$

(8) $$\frac{dc_{P7}}{\mathrm{dt}}=p_{9}c_{P7}^{m}-\left(m_{15}+m_{23}D\right)\cdot c_{P7}$$

(9) $$\begin{array}{ll} \frac{dc_{P5}^{m}}{\mathrm{dt}}= & \frac{g_{23}{}^{g}}{g_{23}{}^{g}+c_{T}{}^{g}}\\ & \times \left(n_{10}\frac{c_{L\mathrm{mod}}{}^{e}}{g_{12}{}^{e}+c_{L\mathrm{mod}}{}^{e}}+n_{11}\frac{c_{P7}{}^{b}}{g_{13}{}^{b}+c_{P7}{}^{b}}\right)\\ & -m_{16}c_{P5}^{m} \end{array}$$

(10) $$\frac{dc_{P5}}{\mathrm{dt}}=p_{10}c_{P5}^{m}-\left(m_{17}+m_{24}D\right)\cdot c_{P5}$$

(11) $$\begin{array}{ll} \frac{dc_{T}^{m}}{\mathrm{dt}}= & \frac{n_{2}}{1+{\left(c_{L}/\left(g_{5}\cdot \left(1+{\left(c_{\mathrm{SnRK}2}/g_{25}\right)}^{j}\right)\right)\right)}^{e}}\\ & \cdot \frac{g_{4}}{g_{4}+c_{\mathrm{EC}}}-m_{5}c_{T}^{m} \end{array}$$

(12) $$\begin{array}{ll} \frac{dc_{T}}{\mathrm{dt}}= & p_{4}\left(c_{T}^{m}+n_{16}\right)\\ & -\left(m_{6}+m_{7}D\right)\cdot c_{T}\left(c_{\mathrm{ZTL}}\cdot p_{5}+c_{\mathrm{ZG}}\right)\\ & -m_{8}c_{T} \end{array}$$

(13) $$\begin{array}{ll} \frac{dc_{E4}^{m}}{\mathrm{dt}}= & n_{15}\cdot \frac{g_{21}{}^{g}}{g_{21}{}^{g}+c_{T}{}^{g}}\cdot \frac{g_{20}}{g_{20}+c_{\mathrm{EC}}}\cdot \frac{g_{6}{}^{e}}{g_{6}{}^{e}+c_{L}{}^{e}}\\ & -m_{34}c_{E4}^{m} \end{array}$$

(14) $$\begin{array}{ll} \frac{dc_{E4}}{\mathrm{dt}}= & p_{23}c_{E4}^{m}-m_{35}c_{E4}-p_{25}c_{E4}c_{E3n}\\ & +p_{21}c_{E34} \end{array}$$

(15) $$\frac{dc_{E3}^{m}}{\mathrm{dt}}=n_{3}\frac{g_{16}{}^{e}}{g_{16}{}^{e}+c_{L}{}^{e}}-m_{26}c_{E3}^{m}$$

(16) $$\begin{array}{ll} \frac{dc_{E3c}}{\mathrm{dt}}= & p_{16}c_{E3}^{m}-m_{9}c_{E3c}c_{\mathrm{COP}1c}-p_{17}c_{E3c}c_{\mathrm{Gc}}\\ & -p_{19}c_{E3c}+p_{20}c_{E3n} \end{array}$$

(17) $$\begin{array}{ll} \frac{dc_{E3n}}{\mathrm{dt}}= & p_{19}c_{E3c}-p_{20}c_{E3n}-p_{17}c_{E3n}c_{\mathrm{Gn}}\\ & -m_{9}c_{E3n}\cdot c_{\mathrm{COP}1d}-m_{10}c_{E3n}\cdot c_{\mathrm{COP}1n}\\ & +p_{21}c_{E34}-p_{25}c_{E4}c_{E3n} \end{array}$$

(18) $$\begin{array}{ll} \frac{dc_{\mathrm{LUX}}^{m}}{\mathrm{dt}}= & n_{13}\cdot \frac{g_{19}{}^{g}}{g_{19}{}^{g}+c_{T}{}^{g}}\cdot \frac{g_{2}}{g_{2}+c_{\mathrm{EC}}}\cdot \frac{g_{6}{}^{e}}{g_{6}{}^{e}+c_{L}{}^{e}}\\ & -m_{34}c_{\mathrm{LUX}}^{m} \end{array}$$

(19) $$\frac{dc_{\mathrm{LUX}}}{\mathrm{dt}}=p_{27}c_{\mathrm{LUX}}^{m}-m_{36}c_{\mathrm{LUX}}-p_{26}c_{\mathrm{LUX}}c_{E34}$$

(20) $$\begin{array}{ll} \frac{dc_{\mathrm{COP}1c}}{\mathrm{dt}}= & n_{5}-p_{6}c_{\mathrm{COP}1c}\\ & -m_{27}c_{\mathrm{COP}1c}\left(1+p_{15}L\right) \end{array}$$

(21) $$\begin{array}{ll} \frac{dc_{\mathrm{COP}1n}}{\mathrm{dt}}= & p_{6}c_{\mathrm{COP}1c}-n_{6}L\cdot c_{P}\cdot c_{\mathrm{COP}1n}\\ & -n_{14}c_{\mathrm{COP}1n}-m_{27}c_{\mathrm{COP}1n}\left(1+p_{15}L\right) \end{array}$$

(22) $$\begin{array}{ll} \frac{dc_{\mathrm{COP}1d}}{\mathrm{dt}}= & n_{14}c_{\mathrm{COP}1n}+n_{6}L\cdot c_{P}\cdot c_{\mathrm{COP}1n}\\ & -m_{31}\left(1+m_{33}D\right)\cdot c_{\mathrm{COP}1d} \end{array}$$

(23) $$\begin{array}{ll} \frac{dc_{\mathrm{EGc}}}{\mathrm{dt}}= & p_{17}c_{E3c}c_{\mathrm{Gc}}-m_{10}c_{\mathrm{EGc}}c_{\mathrm{COP}1c}\\ & -p_{18}c_{\mathrm{EGc}}+p_{31}c_{\mathrm{EGn}} \end{array}$$

(24) $$\begin{array}{ll} \frac{dc_{\mathrm{EC}}}{\mathrm{dt}}= & p_{26}c_{\mathrm{LUX}}c_{E34}-m_{10}c_{\mathrm{EC}}\cdot c_{\mathrm{COP}1n}\\ & -m_{9}c_{\mathrm{EC}}\cdot c_{\mathrm{COP}1d}\\ & -m_{32}c_{\mathrm{EC}}\left(1+p_{24}\cdot L\cdot \frac{c_{\mathrm{Gn}\_\mathrm{tot}}{}^{d}}{g_{7}{}^{d}+c_{\mathrm{Gn}\_\mathrm{tot}}{}^{d}}\right) \end{array}$$

(25) $$\frac{dc_{\mathrm{ZTL}}}{\mathrm{dt}}=p_{14}-p_{12}Lc_{\mathrm{ZTL}}c_{\mathrm{Gc}}+p_{13}c_{\mathrm{ZG}}D-m_{20}c_{\mathrm{ZTL}}$$

(26) $$\frac{dc_{\mathrm{ZG}}}{\mathrm{dt}}=p_{12}Lc_{\mathrm{ZTL}}c_{\mathrm{Gc}}-p_{13}c_{\mathrm{ZG}}D-m_{21}c_{\mathrm{ZG}}$$

(27) $$\frac{dc_{G}^{m}}{\mathrm{dt}}=\frac{g_{17}{}^{g}}{g_{17}{}^{g}+c_{T}{}^{g}}\cdot \left(L\cdot q_{2}\cdot c_{P}+n_{12}\frac{g_{14}}{g_{14}+c_{\mathrm{EC}}}\cdot \frac{g_{15}{}^{e}}{g_{15}{}^{e}+c_{L}{}^{e}}\right)-m_{18}c_{G}^{m}$$

(28) $$\frac{dc_{\mathrm{Gc}}}{\mathrm{dt}}=p_{11}c_{G}^{m}-p_{12}Lc_{\mathrm{ZTL}}c_{\mathrm{Gc}}+p_{13}c_{\mathrm{ZG}}D-m_{19}c_{\mathrm{Gc}}-p_{17}c_{E3c}c_{\mathrm{Gc}}-p_{28}c_{\mathrm{Gc}}+p_{29}c_{\mathrm{Gn}}$$

(29) $$\frac{dc_{\mathrm{ABAR}}^{m}}{\mathrm{dt}}=n_{17}\cdot \frac{g_{24}{}^{g}}{g_{24}{}^{g}+c_{T}{}^{g}}\cdot \frac{c_{L}{}^{e}}{g_{28}{}^{e}+c_{L}{}^{e}}-m_{37}c_{\mathrm{ABAR}}^{m}$$

(30) $$\frac{dc_{\mathrm{PP}2C}}{\mathrm{dt}}=p_{33}\cdot \frac{g_{27}{}^{h}}{g_{27}{}^{h}+c_{\mathrm{AR}}{}^{h}}-m_{39}c_{\mathrm{PP}2C}$$

(31) $$\frac{dc_{\mathrm{SnRK}2}}{\mathrm{dt}}=p_{32}-m_{30}\cdot c_{\mathrm{SnRK}2}\cdot c_{\mathrm{PP}2C}$$

(32) $$\frac{\mathrm{ds}}{\mathrm{dt}}=\left(n_{19}+n_{18}L\right)\cdot \frac{g_{26}{}^{i}}{g_{26}{}^{i}+c_{\mathrm{SnRK}2}{}^{i}}\cdot \left(1-s\right)-m_{29}s$$

$$c_{E34}=p_{25}c_{E4}c_{E3n}/\left(p_{26}c_{\mathrm{LUX}}+p_{21}+m_{9}c_{\mathrm{COP}1d}+m_{10}c_{\mathrm{COP}1n}\right)$$

$$c_{\mathrm{EGn}}=\left(p_{18}c_{\mathrm{EGc}}+p_{17}c_{E3n}c_{\mathrm{Gn}}\right)/\left(m_{10}c_{\mathrm{COP}1n}+m_{9}c_{\mathrm{COP}1d}+p_{31}\right)$$

$$c_{\mathrm{Gn}}=p_{28}c_{\mathrm{Gc}}/\left(p_{29}+m_{19}+p_{17}c_{\mathrm{En}}\right)$$

$$c_{\mathrm{Gn}\_\mathrm{tot}}=c_{\mathrm{Gn}}+c_{\mathrm{EGn}}$$

$$c_{\mathrm{AR}}=0.5\cdot \left(A_{0}+c_{\mathrm{ABAR}}^{m}+g_{29}-\sqrt{\left(A_{0}+c_{\mathrm{ABAR}}^{m}+g_{29}\right)-4A_{0}c_{\mathrm{ABAR}}^{m}}\right)$$

Where $c_{i}^{m}\;\mathrm{and}\;c_{i}$ stand for dimensionless concentrations of mRNA and protein, respectively. The time unit is an hour. Index “*i*” labels the molecular components as follows:

*ABAR* ABAR mRNA

*AR* ABA complex with ABAR protein

*COP1c* COP1 cytoplasmic protein

*COP1d* COP1 nuclear protein, day

*COP1n* COP1 nuclear protein, night

*E3* ELF3 mRNA

*E3c* ELF3 cytoplasmic protein

*E3n* ELF3 nuclear protein

*E4* ELF4 mRNA and protein

*E34* nuclear protein complex ELF3-ELF4

*EC* nuclear protein complex ELF4-ELF3-LUX

*EGc* cytoplasmic protein complex ELF3-GI

*EGn* nuclear protein complex ELF3-GI

*G* GI mRNA

*Gc* GI cytoplasmic protein

*Gn* GI nuclear protein

*Gn_tot* total amount of GI protein in nucleus

*L* LHY/CCA1 mRNA and protein

*Lmod* LHY/CCA1 modified protein

*Ltot* total amount of LHY/CCA1 protein

*LUX* LUX mRNA and protein

*P5* PRR5 mRNA and protein

*P7* PRR7 mRNA and protein

*P9* PRR9 mRNA and protein

*PP2C* PP2C active protein

*SnRK2* SnRK2 active protein

*T* TOC1 mRNA and protein

*ZG* cytoplasmic protein complex GI-ZTL

*ZTL* ZTL protein

Symbol “s” corresponds to stomata aperture in relative units (the maximum of s is equal to 1). A_0_ is the total ABA level in relative units. ABA level for most of calculations was set to 1, unless it is stated otherwise (ABA_0_ = 1).

The quasi-steady state approximation for the nuclear complexes ELF3-GI, ELF3-ELF4 and nuclear GI protein, which reduces the number of equations, was taken from the P2011 model. COP1 nuclear activity was described by two forms of COP1 ([2], eqs. 20, 21). The parameters *n*_*j*_ represent the rate constants of transcription and stomata opening, *m*_*j*_ are the rate constants of degradation; *p*_*j*_ are constants of translation, protein modification and protein complex formation; *g*_*j*_ are Michaelis-Menten constants and *a, b, c, d, e,f, g, h, i, j* are Hill coefficients; *q*_*j*_ are the rate constants of acute (P-dependent) light activation of transcription. The acute light response in activation of *PRR9, LHY/CCA1, GI,* transcription (eqs. 5, 1, 27) was modelled using a light-sensitive activator – protein P (*c*_*P*_), which is accumulated in darkness and was degraded in light similarly to the P2011 model (eq. 4). L = 1 when light is present, 0 otherwise; D = 1-L. The*L*(*t*) function was used to simulate smooth transitions between L and D analogous to the P2011 model:

$$\begin{array}{l} L\left(t\right)=0.5\cdot ((1+\tanh((t-24\cdot \mathrm{floor}(t/24)-\mathrm{dawn})/T))-(1+\tanh((t-\\ 24\cdot \mathrm{floor}\left(t/24\right)-\mathrm{dusk})/T))+(1+\tanh((t-24\cdot \mathrm{floor}(t/24)-24)/T))) \end{array}$$

Where *dawn* and *dusk* are the phases of dawn and dusk (normally *dawn = 0*); T is the duration of twilight (we used T = 0.05 h); tanh and floor – standard functions of hyperbolic tangent and rounding operation. This representation of periodic input corresponds to the Input Signal Step Function, which now has convenient software support in SBML [14]. Mutations of the clock genes were simulated by decreasing the rate of transcription of the corresponding gene to zero. *TOC1-ox* plants were modelled by adding the background activation of TOC1 translation (parameter n16 in eq. 12; n16 = 0 for wild type).

The equations for COP1 E3 ligase activities (eqs. 20, 21), which were developed in P2011, are independent of the clock and their parameters were constrained from the data on the kinetics of COP1 substrates HFR1 and HY5 proteins upon dark/light transitions similarly to P2011 [15]. The equations for HY5 and HFR1 proteins, which were used only for the optimization of COP1 parameters, are taken from P2011:

$$dc_{\mathrm{HY}5}/\mathrm{dt}=p_{22}-m_{38}c_{\mathrm{HY}5}c_{\mathrm{COP}1d}-m_{25}c_{\mathrm{HY}5}c_{\mathrm{COP}1n}$$

$$dc_{\mathrm{HFR}1}/\mathrm{dt}=p_{30}-m_{28}c_{\mathrm{HFR}1}c_{\mathrm{COP}1n}$$

2. Modelling of ABA signalling and its relation with the clock

To model the mutual connections between the clock and ABA signalling, we extended the clock model by including ABA-induced reactions.

The first group of reactions of ABA signalling is related with the inactivation of protein phosphatase PP2C (protein phosphatase of 2C type) by the ABA complex ([11]; Figure 1B). Based on the data showing relatively small diurnal changes in ABA concentration in plants [10,16], we assumed that ABA level is constant in our model and treated it as a parameter ABA_0_. Next we included the potential ABA receptor ABAR, which is a necessary component of the regulation of both *TOC1* expression and stomatal aperture by ABA [9]. *ABAR* expression has a strong circadian pattern, peaking in the morning [9]. Our analysis of the *ABAR* promoter in Arabidopsis revealed 2 CCA1 binding sites (CBS) AAATCT [17], in addition to the known TOC1-binding sites [3,9], suggesting a possible mechanism for the regulation of *ABAR* transcription by the clock. This was described in our model through activation of *ABAR* expression by LHY/CCA1 and its inhibition by TOC1 ([3,17], eq. 29). To simplify the model and reduce the number of equations, we used a quasi-steady state approximation for the ABA complex with ABAR, assuming that ABAR protein level follows the cognate mRNA. PP2C activity was described through its inhibition by this ABA complex (eq. 30).

The second important group of reactions of ABA signalling is related with the activation of SNF1 (Sucrose-Nonfermenting Kinase1)-related protein kinase SnRK2 by ABA [11]. This activation happens through double-negative interactions: the ABA complex inactivates PP2C and PP2C inactivates SnRK2. So we included in the model an equation for SnRK2 activity, which is inhibited by PP2C (eq. 31). After activation by ABA, SnRK2 mediates multiple reactions of the ABA pathway, including induction of transcription of multiple target genes which possess ABRE elements (ABA-responsive elements) in their promoters [11]. As *TOC1* was reported to be induced by ABA [9], we analysed the *TOC1* promoter and identified 2 ABRE elements. This suggested that a mechanism of *TOC1* induction by ABA is similar to other genes and mediated by SnRK2 (Figure 1B). Therefore we included in the model the activation of *TOC1* expression by SnRK2, assuming that this activation competes with inhibition of *TOC1* by LHY/CCA1 protein ([9], eq. 11). An analogous mechanism of ABA signalling through the ABA/PP2C/SnRK2 pathway is involved in the acceleration of stomatal closing by ABA [11], which we included into the model. The equation for stomatal aperture describes stomatal opening in a phenomenological way, which allowed us qualitatively compare the model behaviour with physiological data. Additionally to the inhibition of stomatal opening by ABA signalling through active SnRK2, we included the known activation of stomatal opening in the presence of light ([18], eq. 32).

So finally the clock model was extended by including eqs. (29)-(32) for *ABAR*, PP2C, SnRK2 and stomata aperture (variable “s”). Thus we connected the clock with a simple version of ABA signalling, which enabled us to simulate *TOC1* and stomatal regulation by ABA. The effective parameters of the ABA-related reactions were optimized together with other parameters the clock model against multiple perturbations as described below.

Additional file 1: Figure S1 shows simulated diurnal kinetics of the main components of ABA signalling under the optimal parameters, which are presented in Additional file 2: Table S1. The peak of *ABAR* transcription soon after dawn corresponds to the publicly available microarray data (http://diurnal.mocklerlab.org/), which agrees with RT-Q-PCR analysis [9]. The accumulation of ABA-ABAR complex in the day time leads to inactivation of PP2C and activation of SnRK2, with the peak of SnRK2 activity in the afternoon (Additional file 1: Figure S1). SnRK2 then induces *TOC1* expression and stimulates the closing of stomata as described above.

3. Solving the system of ODEs

The extension of P2011 by adding newly discovered processes of the inhibition of the *PRR9, PRR7, PRR5, LUX, ELF4* and *GI* clock genes by TOC1 (Figure 1A) resulted in the addition of 7 new parameters to P2011. Our model was also extended compared to P2011 by adding reactions describing ABA-related processes (Figure 1B), which introduced 14 more new parameters. Finally, the model describes the dynamics of stomatal opening, which was absent from P2011, resulting in 5 more new parameters. Therefore, the higher level of biological complexity of the model and its extension to hormonal signalling through ABA inevitably increased the parameter space, adding 26 parameters compared to P2011. This allowed our model to describe experimental data that were not described by P2011, such as the inhibition of multiple clock genes by TOC1, stimulation of *TOC1* expression by ABA signalling, changing the clock period by ABA and the dynamics of stomata presented in the Results below. In addition, the new structure of the clock, which now incorporates ABA signalling to the clock through TOC1 (Figure 1A) allowed us to improve the description of the data on the clock kinetics compared to P2011, as shown in the Results below.

Parameter values were either constrained based on experimental data or fitted to multiple time-series data sets, similarly to the P2011 model. 49 out of the total 133 parameters were constrained based on the available experimental data. Most of these parameters have the same values as in P2011 because they were constrained by the same data. Other parameters were fitted to multiple datasets similarly to P2011, but their values may be different from P2011 because the structures of the models are different. The optimal set of parameters is presented in Additional file 2: Table S1. The new model, under the optimal parameter values, retains most of its properties from P2011, keeping the good fit to data from various conditions, such as diurnal cycles of the wild type and *lhy/cca1*, *lhy/cca1/gi* and *elf3* mutants, as well as continuous light or darkness in the wild type and multiple mutants. For example, the period values for the simulated free-running clock in wild type and mutant plants were 24.5 h for wild type under constant light conditions, 26.6 h for wild type under constant darkness and 17.7 h, 21.4 h, 30.1 h, 30.7 h, 21.2 h for *lhy/cca1*, *toc1*, *ztl*, *prr79* and *gi* mutants in constant light, which match the experimental observations [8,19-22]. Additionally *lhy/cca1/gi*, *elf3, elf4* and *lux* mutants were arrhythmic in constant light, in agreement with the data [8,23-25]. We also tested the sensitivity of the model to the variations of the newly introduced parameters of the inhibition of target genes by TOC1 and ABA signalling. Additional file 1: Figure S2 shows the relative changes in the amplitude of *LHY* expression and the clock period in constant light conditions under 10% changes of each parameter. We observed less than 3% changes in the period and less than 12% change in the amplitude of *LHY* mRNA under variations of these parameters, which shows that the extended model retains its robustness to parameter perturbations from P2011 (Additional file 1: Figure S2). Thus, the optimal set of parameters demonstrated good correspondence to multiple datasets and provides robust behaviour in the model. However, given the significant complexity of the model, which is related to the complexity of the biological processes involved, and the number of unknown parameters, we cannot exclude the existence of other parameter sets that might equally describe the data. The detailed study of this large parameter space lies outside the scope of this paper. The model with the current parameter set matches well enough to the data to make it a useful instrument for the exploration of the possible mechanisms of the observed biological phenomenon.

ABA modulates expression of the clock genes through the induction of *TOC1* expression followed by suppression of TOC1 target genes. Additional file 1: Figure S3 shows the dependence of the peak mRNA values of all clock genes on the ABA levels, demonstrating that *ELF4, LUX, GI, PRR7* and *PRR5* are the most sensitive to ABA in our model.

### *Modulation of circadian dynamics* via *clock gene repression by TOC1*

**Figure 3:**
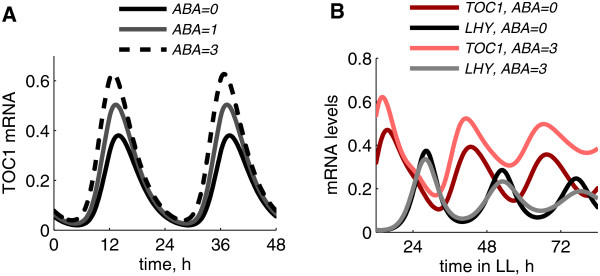
**Modulation of clock dynamics by ABA.** Diurnal profiles of *TOC1* mRNA (**A**) under various levels of ABA (ABA level is expressed in relative units; the wild-type level is 1). Simulations were run under a 12 L:12D diel cycle. **B**: Free running oscillation of *LHY/CCA1* and *TOC1* mRNA under constant light conditions in the absence or presence of ABA. The 1.3 h period lengthening in the presence of increased ABA in the model simulation is close to the reported 1.5 h lengthening of the period of the clock marker *CAB2: LUC* under constant light conditions [29].

**Figure 4:**
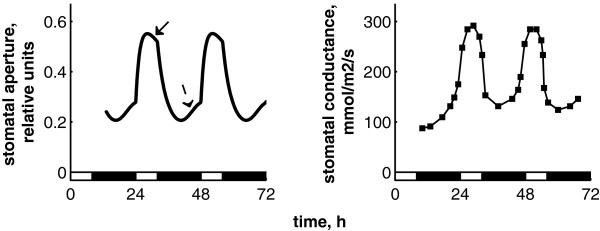
**The kinetics of stomata opening in 8 L:16D diel cycles in the model (A) and in the experimental data (B), redrawn from **[18]**.** Zero time corresponds to dawn. Solid and dashed arrows on A show the “dusk” and “dawn” anticipations respectively as described in the text.

The extension of our previous P2011 model with the parallel suppressive effects of TOC1 on the oscillator genes (Figure 1A; [3]) allowed us to improve the description of data from several clock mutants compared to P2011, without affecting the good fit of the P2011 model to the data of other mutants. Figure 2 shows the profiles of clock gene expression in the simulated *TOC1*-mis-expressing plants and explains the mechanisms involved. The observed decrease in the peak level of *LHY/CCA1* expression in the *toc1* mutant suggested that TOC1 was an activator of *LHY* and *CCA1*[4]. Although TOC1 is a repressor of *LHY/CCA1* in the model, removing TOC1 also reduces *LHY/CCA1* expression (Figure 2A). The simulated *toc1* mutation increases the levels of the remaining *LHY/CCA1* inhibitors, the PRR (PSEUDO-RESPONSE REGULATOR) proteins (PRR9, PRR7, PRR5), by relieving the negative regulation of *PRR* transcription by TOC1 (Figure 2C). In the model, this results in instant down-regulation of *LHY/CCA1* mRNA after switching from a simulated light/dark cycle to constant light (LL) conditions. Figure 2A demonstrates that the simulated *toc1* mutant has *LHY/CCA1* at 0.85-fold the peak level in wild type after two days in LL, which is close to the experimentally observed 0.82-fold for *LHY* mRNA (Figure 2B, [4,26]). In contrast, the *toc1* mutant simulated in the P2011 model initially contradicts the data, as it first shows an increase of *LHY/CCA1* levels followed by only a slight decrease (0.97-fold after two days of LL), and through a more indirect mechanism [2].

Over-expression of *TOC1* also resulted in a lower level of *LHY/CCA1* expression, which is due to the direct suppression of *LHY/CCA1* expression by the increased level of the repressor, TOC1 (Figure 2A). This effect of TOC1 was present already in the P2011 model. However, the P2011 model failed to describe the longer period of the TOC1-overexpressor line (*TOC1-ox*) under constant light, which was observed in the data [27]. The discrepancy in the earlier model was caused by increased levels of the PRR inhibitors as well as TOC1, which resulted in a reduction of the *LHY/CCA1* level. This led to a shortening of the period in the simulated *TOC1-ox* lines in P2011 rather than a longer period. The current model describes the experimentally observed prolongation of the period due to delay in *LHY/CCA1* in *TOC1-ox* compared to the wild type because repression by TOC1 reduces the level of the other PRRs (Figure 2C). The simulated *TOC1-ox* in Figure 2A has a 1.5 h longer period than the wild type. The period difference between the *TOC1-ox* and wild type increases with the increase in the level of *TOC1* expression in our model, in agreement with the data [21,27]. For example, 3-fold increase of the *TOC1* overexpression level compared to the level shown in Figure 2A results in 5 h longer period of the *TOC1-ox* compared the wild type (while it has 1 h shorter period in P2011). The large prolongation of the *TOC1-ox* period (5 h) corresponds to the experimental observations for the *TOC1-ox* lines with high overexpression levels of *TOC1*[27]. The oscillations are damping in *TOC1-ox* in our model simulations (Figure 2A) similarly to the data [27], to the point of the loss of rhythmicity of the simulated *TOC1-ox* at very high levels of TOC1-overexpression, which also agrees with the data [21]. The model suggests that the period increase is due to the simultaneous up-regulation of TOC1 and down-regulation of the other *PRR* expression in the *TOC1-ox* lines, which allows a longer interval of *LHY/CCA1* expression than in the wild type.

Figure 2C shows schematically the mechanisms of regulation of *LHY/CCA1* expression by the wave of PRR inhibitors in lines with different levels of TOC1: In the *toc1* mutant, increased PRR levels reduce *LHY/CCA1* levels, while the absence of TOC1, the last inhibitor, shortens the period. In *TOC1-ox*, *LHY/CCA1* expression is repressed mainly by TOC1 protein, which declines when *LHY* is raising (LHY and TOC1 are anti-phasic) and thus allowing oscillations to occur but with longer period than in wild type (Figure 2A). Thus the model offers an explanation of the low *LHY* and *CCA1* levels in the *toc1* mutant [4] that is consistent with the biochemical data showing that TOC1 is a repressor [3,7]. The mechanism depends upon the inhibition of multiple clock genes by TOC1, particularly the *PRR*s (Figure 2C).

Additionally to affecting the expression of the morning genes *LHY/CCA1, PRR9, PRR7, PRR5*, TOC1 negatively regulates the evening genes *LUX (LUX ARRHYTHMO), ELF4 (EARLY FLOWERING 4)* and *GI (GIGANTEA)* in the experimental data [3] and in our model. This is in clear contrast with the P2011 model, where TOC1 affected only *LHY/CCA1* expression directly. The new model suggests that increased levels of evening gene expression result in the arrhythmia of the *lhy/cca1/toc1* triple mutant under constant light conditions, as opposed to the damped oscillation of the *lhy/cca1* double mutant. This agrees with experimental observations [28].

### TOC1-mediated effects of ABA on circadian timing

Figure 3A shows simulated diurnal profiles of *TOC1* expression under increasing ABA levels, which might correspond to increasing levels of stress, such as drought [9,10]. In agreement with the data [9], ABA accelerates *TOC1* expression in the afternoon. The up-regulation of TOC1 under higher ABA levels resulted in the suppression of expression of TOC1 target genes in our simulations (Additional file 1: Figure S3), which agrees with the data [3]. The model also suggests that the up-regulation of *TOC1* by ABA should lengthen the circadian period (1.3 h) under free-running conditions (Figure 3B), similar to the *TOC1-ox* line (Figure 2). This agrees with the experimental observations, showing periods about 1.5 h longer than WT in the expression of the clock output gene *CAB2* in plants treated with high exogenous ABA concentrations [29].

Altogether, the extension of the P2011 model including the widespread repression of core components by TOC1 (Figure 1A) and the inclusion of the regulatory function of ABA on *TOC1* expression allowed us to improve the description of multiple datasets on the clock kinetics in wild type and mutant plants, and suggest mechanisms to explain previously paradoxical data.

### Gating of ABA signalling by the clock

**Figure 5:**
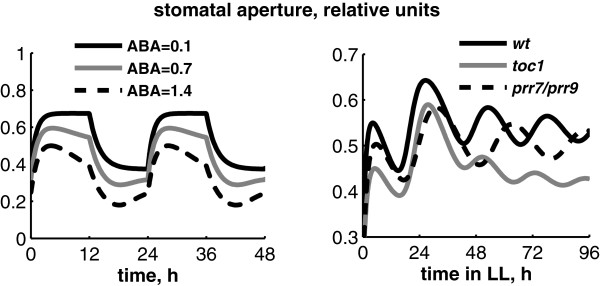
**Simulated kinetics of stomata opening and its regulation by ABA and the circadian clock. A**: Diurnal profiles of stomata aperture of wild type plants under 12 L:12D conditions for various ABA levels; **B**: Free running rhythms of stomata aperture under constant light conditions, calculated for wild type, *toc1* and *prr7/prr9* mutants.

We next examined the biological relevance of the interactions between ABA and the circadian clock. To that end, we extended the model to the regulation of an important physiological process downstream of the clock - stomatal aperture. The equation for stomatal aperture describes the regulation of stomatal dynamics by light and by ABA signals [10,18]. The simulated diel kinetics of stomatal aperture are shown on Figure 4, together with data from wild-type Arabidopsis plants in the same conditions [18]. Light activation results in a more open state of stomata during the day than in the night, in agreement with the experimental data [18]. Stomatal dynamics also have a pronounced diurnal pattern related to ABA signalling as discussed below. Figure 5A shows simulated stomatal dynamics under different ABA levels. Increased ABA levels correspond to drought conditions, which lead to a more closed state of stomata, in agreement with the data [10]. The closing of stomata by ABA is a well-known physiological response in plants, which is important for the reduction of transpiration under drought [10,11]. Additionally to the modulation of the average daily size of the stomatal pore, ABA regulates the peak and trough phases of stomatal aperture. In the model, ABA signal is gated by the clock through *ABAR* transcription in the morning and the following gradual increase of SnRK2 (SNF1/Sucrose-Nonfermenting Kinase1-related protein kinase) activity during the day (Figure 1B, [11]). The SnRK2 kinase in turn regulates multiple downstream processes including the dynamics of stomatal aperture [11], as described in the Methods. Figure 5A demonstrates that the resulting diurnal timecourse of stomata aperture is characterized by a morning peak with some decline towards dusk –“dusk anticipation”, resulting from higher sensitivity of stomata to ABA in the afternoon. Similarly, stomata are less sensitive to ABA before dawn, which results in an increased stomatal aperture before dawn – “dawn anticipation”. Both dawn and dusk anticipations agree with the data [10] and are important for closing stomata in the heat of the afternoon to reduce a water loss and opening of stomata in the cool of the morning to prepare plants for photosynthesis after dawn [30]. The modelling here allowed us to describe the data on stomatal dynamics and showed that circadian regulation of ABAR transcription was consistent with the gating of the ABA signalling by the circadian clock and the resulting rhythmic stomatal dynamics.

**Figure 6:**
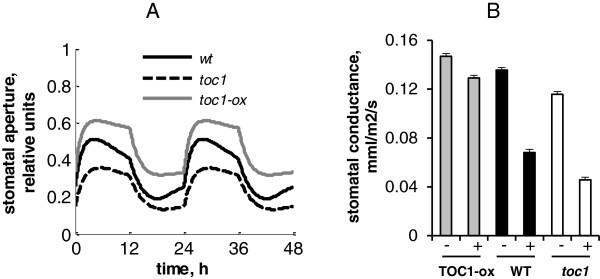
**Effect of TOC1 on stomata size. A**: Simulated time courses of stomata aperture in wild type, *TOC1-ox* and *toc1* mutant under 12 L:12D cycle for ABA = 1.3. *TOC1-ox* was simulated at n16 = 0.3. **B**: Experimental data on stomata conductance in wild type, *TOC1-ox* and *toc1* mutant in absence (-) and presence (+) of 5 μM of exogenous ABA. Data are redrawn from [9].

The circadian regulation resulted in distinctive, free-running rhythms of stomatal opening under constant light conditions for simulated wild type and mutant plants (Figure 5B), similar to those observed experimentally [18]. The period of this rhythmic clock output corresponded to the one of the clock itself in our simulations, in agreement with data [18]. Figure 5B demonstrates this for the short- and long-period mutants *toc1* and *prr7/prr9*. Additionally to the change in period length, the simulated *toc1* mutant had smaller stomatal aperture compared to wild type (Figure 5B). To explore the effect of TOC1 on stomata in more detail, we compared the dynamics of stomata in wild type, *toc1* mutant and *TOC1-ox* lines. Figure 6A demonstrates that the simulated stomata were more open in the *TOC1-ox* and more closed in the *toc1* compared to wild type, which fully agree with the experimental observations [9]. This effect was due to the direct inhibition of *ABAR* expression by TOC1. Simulation of the model showed that this effect was especially pronounced under high ABA levels (Figure 6B, [9]). Additionally, the model suggests that TOC1 regulation will alter the diurnal gating of the stomatal response to ABA, because both simulated *TOC1-ox* and *toc1* mutant lines had less pronounced anticipation of dawn and dusk (Figure 6A).

## Conclusions

The revised clock model explains clock gene profiles of mutants with altered *TOC1* expression, resulting from the pervasive transcriptional repression by TOC1. The model was extended to explore the mutual connections between TOC1 targets and ABA signalling, resulting in downregulation of clock gene expression and lengthening of the free running period under high ABA conditions. Moreover, TOC1’s participation in the rhythmic gating of ABA signalling can explain well-characterised rhythms in physiological processes downstream of ABA, such as the regulation of stomata aperture. The clock effects on stress signalling are significant at the whole-plant level, as plant survival under dry conditions was reduced in *TOC1*-ox plants and increased in *toc1* mutants [9]. Moreover, the close overlap between TOC1- and ABA-regulated genes in transcriptome profiles [9] suggests that other ABA-mediated stress responses are similarly responsive to TOC1. Integrating such physiological response pathways, even if they are represented in a simplified way, with the clock model will allow to expand our knowledge on the temporal regulation of plant physiology in its normal context, the unavoidable day-night cycle.

## Methods

The system of ordinary differential equations was solved using MATLAB, integrated with the stiff solver ode15s (The MathWorks UK, Cambridge). The SBML version of the model will be available upon publication from the Biomodels database [31] (accession number BIOMD0000000445) and the Plant Systems Modelling portal (http://www.plasmo.ed.ac.uk). A MATLAB version of the model is available from the authors upon request.

## Abbreviations

LHY: LATE ELONGATED HYPOCOTYL; CCA1: CIRCADIAN CLOCK ASSOCIATED 1; PRR9: PRR7, PRR5, PSEUDO-RESPONSE REGULATORs 9, 7, 5; TOC1: TIMING OF CAB EXPRESSION 1; GI: GIGANTEA; ZTL: ZEITLUPE; ELF3: EARLY FLOWERING 3; ELF4: EARLY FLOWERING 4; LUX: LUX ARRHYTHMO; COP1: CONSTITUTIVE PHOTOMORPHOGENIC 1; EC: Evening complex; ABA: Abscisic acid; ABAR: ABA receptor (magnesium-chelatase H subunit); ABRE: ABA-responsive elements on gene promoters; PP2C: Protein phosphatase of 2C type; SnRK2: SNF1 (Sucrose-Nonfermenting Kinase1)-related protein kinase; 12 L:12D: 12 h light/12 h dark daily cycle of light.

## Competing interests

The authors declare that they have no competing interest.

## Authors’ contributions

AP, PM, AJM designed the model and drafted the manuscript. AP performed the simulations. All authors read and approved the final manuscript.

## Supplementary Material

Additional file 1: Figure S1 The profiles of the main components of ABA signalling, simulated under 12 L:12D diel cycles. The daily changes in the levels of ABA-ABAR, active PP2C and SnRK2 are shown by black, blue and red lines, respectively. **Figure S2.** The change in the relative amplitude and period of *LHY* mRNA in constant light conditions measured under 10% increase and decrease of the model parameters of inhibition of target genes by TOC1 and ABA signalling to the clock. **Figure S3.** Relative changes in the peak expression levels of the clock genes at varied ABA values. Simulations were done under 12 L:12D conditions. Expression levels were normalized to the value in absence of ABA. Click here for file

Additional file 2: Table S1 Optimal set of the model parameter values. Click here for file
