# Supplementary material for: Modelling the widespread effects of TOC1 signalling on the plant circadian clock and its outputs
Source: BMC Syst Biol. 2013 Mar 19;7:23. doi: 10.1186/1752-0509-7-23 (PMC3614443; doi:10.1186/1752-0509-7-23)
Supplement: Additional file 1: Figure S1 — The profiles of the main components of ABA signalling, simulated under 12 L:12D diel cycles. The daily changes in the levels of ABA-ABAR, active PP2C and SnRK2 are shown by black, blue and red lines, respectively. Figure S2. The change in the relative amplitude and period of LHY mRNA in constant light conditions measured under 10% increase and decrease of the model parameters of inhibition of target genes by TOC1 and ABA signalling to the clock. Figure S3. Relative changes in the peak expression levels of the clock genes at varied ABA values. Simulations were done under 12 L:12D conditions. Expression levels were normalized to the value in absence of ABA. [file 1752-0509-7-23-S1.pptx]

## Slide 1
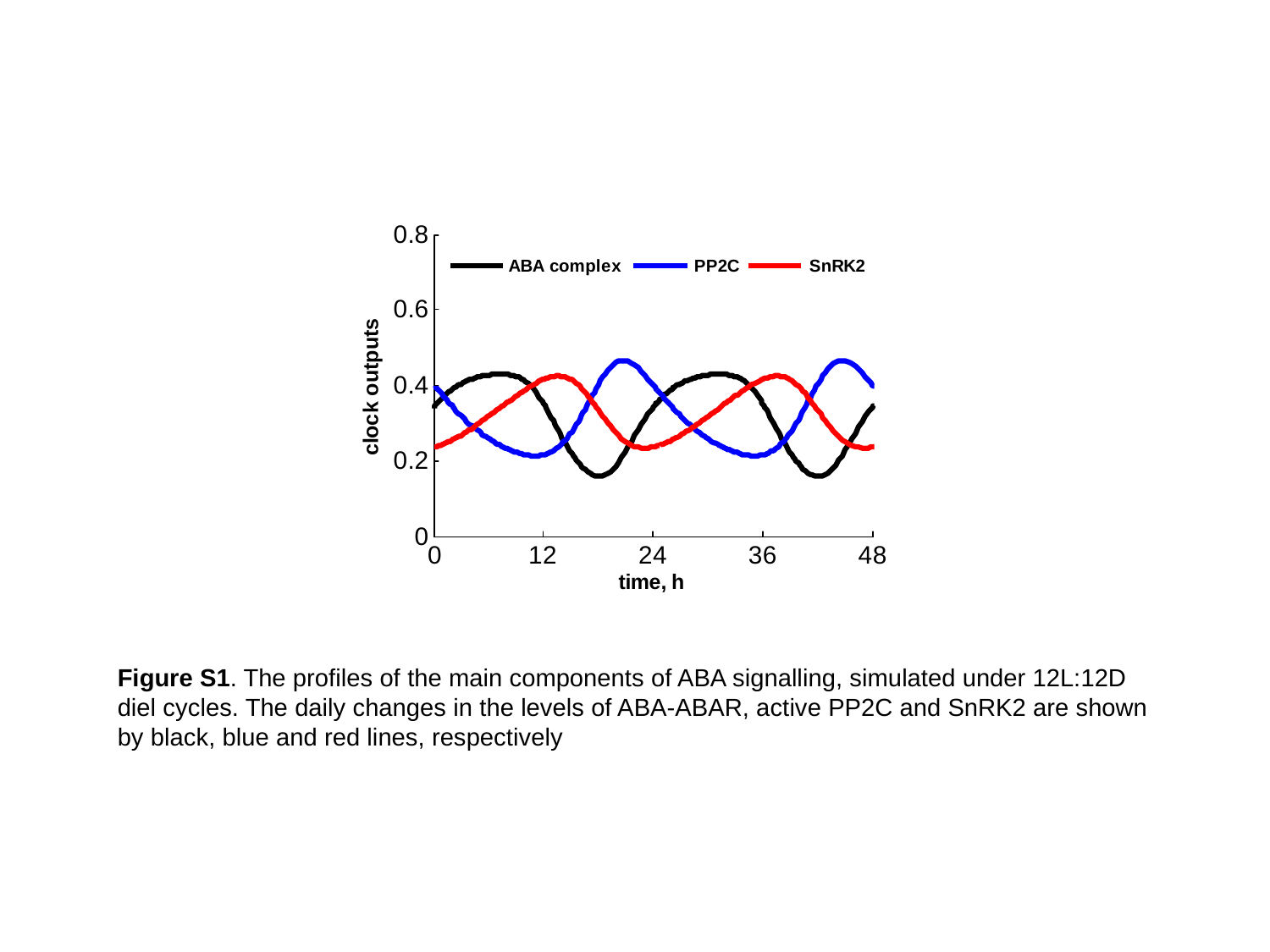

Figure S1. The profiles of the main components of ABA signalling, simulated under 12L:12D diel cycles. The daily changes in the levels of ABA-ABAR, active PP2C and SnRK2 are shown by black, blue and red lines, respectively

## Slide 2
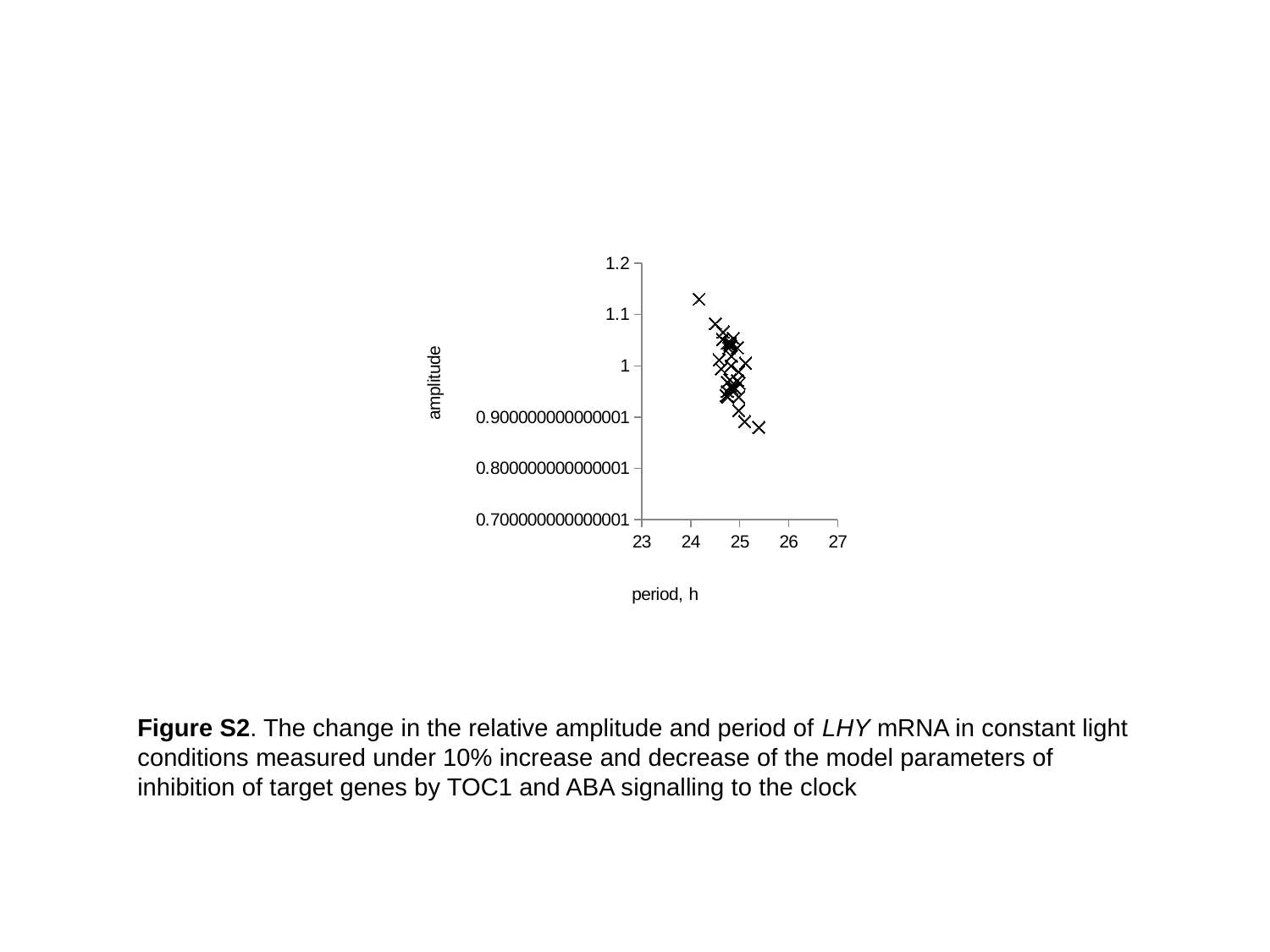

### Chart
| Category | |
|---|---|Figure S2. The change in the relative amplitude and period of LHY mRNA in constant light conditions measured under 10% increase and decrease of the model parameters of inhibition of target genes by TOC1 and ABA signalling to the clock

## Slide 3
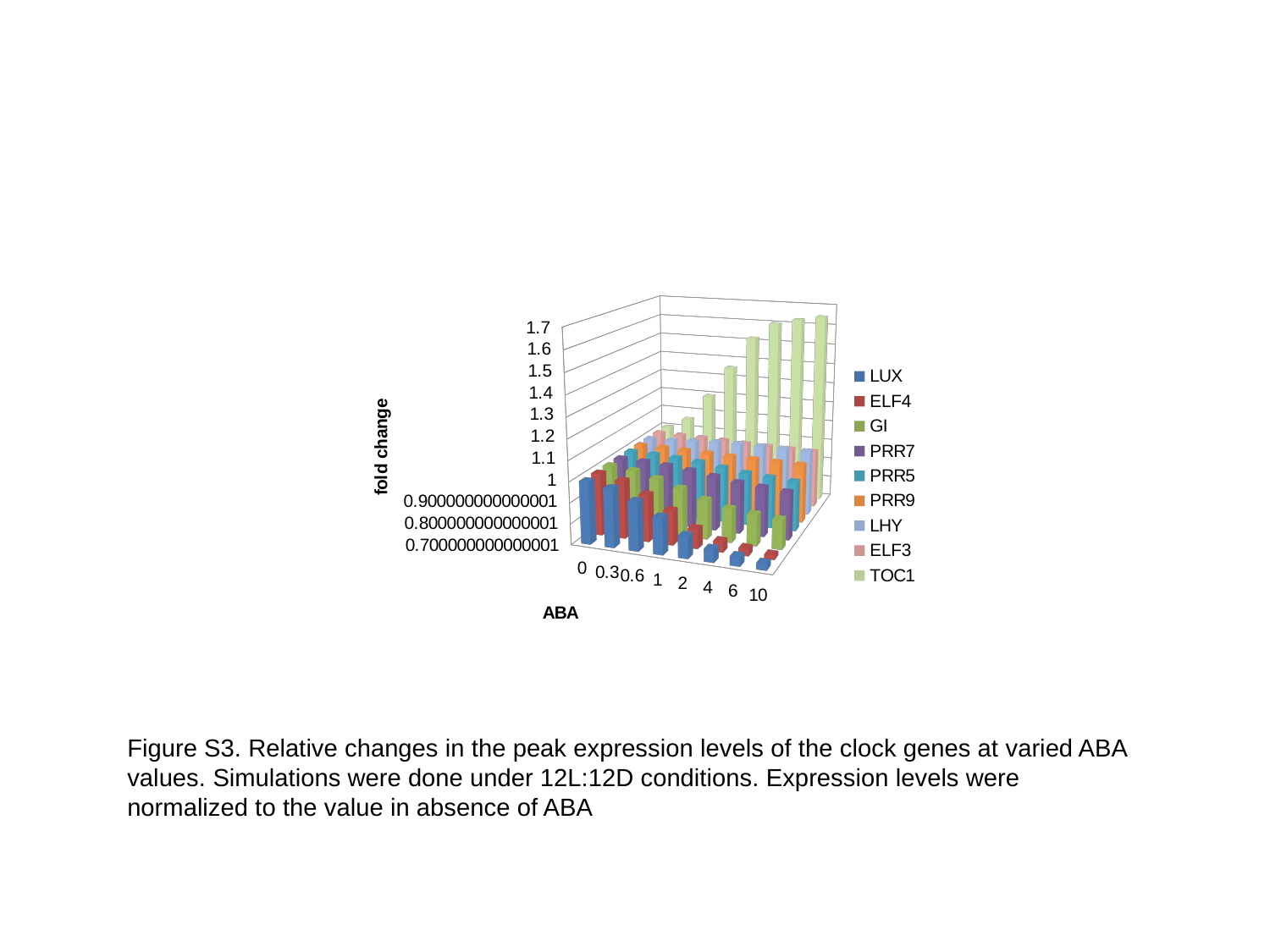

[unsupported chart]
Figure S3. Relative changes in the peak expression levels of the clock genes at varied ABA values. Simulations were done under 12L:12D conditions. Expression levels were normalized to the value in absence of ABA
